# Thromboelastogram changes are associated with postoperative complications after cytoreductive surgery

**DOI:** 10.1515/pp-2023-0018

**Published:** 2024-10-18

**Authors:** Noam Goder, Lilach Zac, Nadav Nevo, Fabian Gerstenhaber, Or Goren, Barak Cohen, Idit Matot, Guy Lahat, Eran Nizri

**Affiliations:** Department of Surgery B, Peritoneal Surface Malignancy and Melanoma Unit, Tel-Aviv Sourasky Medical Center, Tel Aviv, Israel; Division of Anesthesia, Intensive Care, and Pain Management, Tel-Aviv Sourasky Medical Center, Tel Aviv, Israel; Sackler Faculty of Medicine, Tel-Aviv University, Tel-Aviv, Israel

**Keywords:** thromboelastogram, hyperthermic intraperitoneal chemotherapy, postoperative complications

## Abstract

**Objectives:**

Cytoreductive surgery (CRS) and hyperthermic intraperitoneal chemotherapy (HIPEC) is used to treat peritoneal surface malignancies. However, surgical morbidity is high, and prediction of severe postoperative complications (SPC) is limited. We hypothesized that the changes in thromboelastogram (TEG) values following CRS could be associated with SPC.

**Methods:**

We retrospectively analyzed a cohort of CRS and HIPEC patients who had TEG measured before and after CRS. Clinical and postoperative data were retrieved from a prospectively maintained database.

**Results:**

Our 37-patient cohort was comprised of 24 men and 13 women with an age (median, [interquartile range, IQR]) 55 (47–65) years, of whom six had SPC. The ones with SPC did not differ from the others in age, sex, tumor histology or preoperative chemotherapy. The extent of surgery as measured by the peritoneal carcinomatosis index and the number of organs resected was comparable between SPC group vs. no SPC [9 (3–10.5) vs. 9 (5–14), p=1.0; 2 (0.75–2.25) vs. 2 (1–3), p=0.88, respectively]. The TEG parameters showed increased R- and K- time for the patients with SPC compared to those without (6 ± 3.89 vs. 4.05 ± 1.24, p=0.01; 1.65 ± 0.63 vs. 1.25 ± 0.4, p=0.03, respectively). The TEG values were significantly associated with SPC in the multivariable analysis (odds ratio=1.53, p=0.05).

**Conclusions:**

TEG changes are associated with SPC. Intra-operative markers of SPC could guide intraoperative decisions, such as stool diversion and postoperative triage of patients to an appropriate level of care.

## Introduction

Cytoreductive surgery (CRS) combined with heated intra-peritoneal chemotherapy (HIPEC) is an established surgical procedure for the treatment of peritoneal metastases from various primary tumors (such as appendiceal [1], colonic [2], ovarian [3], gastric [4] and primary peritoneal [5]). Although long-term survival can be achieved in well-selected patients operated by experienced teams, complication rates are high, with major morbidity rates ranging from 18 to 52 % and mortality rates from 0 to 8 % [6, 7]. Procedure- and patient-related factors were implicated in the high complication rate of CRS+HIPEC. Since the aim of CRS is to resect all of the macroscopic disease, it often entails multivisceral resection with its associated morbidity. The addition of HIPEC may further increase morbidity [8], although its contribution to the complication rate is probably minor in comparison to CRS itself. Patient condition, including performance status, medical comorbidities, and tumor burden and origin are all related to the major morbidity rate, and preoperative patient selection is crucial for improving surgical outcomes. Intraoperatively, use of stool diversion was suggested to minimize the effect of hollow viscus anastomotic leakage [9]. However, there is no robust marker of postoperative complications, and risk assessment relies on clinical (performance status and comorbidities) and surgical (number of organs resected, surgical complexity, etc.) parameters, similar to other major surgeries [10].

The inflammatory response is associated with coagulation processes. The inflammatory stimulus induced by surgery is comprised of exposure of tissue factor (TF) due to damage to blood vessels, and of the release of damage-associated molecular patterns, including intracellular proteins to the systemic circulation. All of these factors are known to modulate the coagulation process [11]. Thus, measuring coagulation parameters may provide information on the inflammatory response inflicted by the surgery. Thromboelastography (TEG) provides information about the coagulation and fibrinolysis phases of the coagulation process, which encompasses the interactions of various blood components (platelets, coagulation factors and leukocytes). CRS+HIPEC is associated with a transfusion requirement that is greater than other major surgeries due to the large dissection planes associated with the peritonectomies and multivisceral resections [12, 13], therefore coagulation function is routinely monitored during those surgeries [14].

The aim of this study was to evaluate whether TEG parameters, as measured at the beginning of the operation and again after completion of CRS (before HIPEC), are correlated to the inflammatory response induced by the surgery, and their association with severe postoperative complications (SPC).

## Patients and methods

### Patients

This is a retrospective analysis of a prospectively maintained institutional database, which contains data on patient characteristics, pre- and postoperative treatments, tumor characteristics and long-term outcomes. Between February 2015 and January 2023, a total of 122 surgeries involving CRS and HIPEC for peritoneal metastases were performed at our institution, with TEG conducted as detailed below in 37 cases. Significantly, for the majority of this period, there was no dedicated anesthesia team for CRS and HIPEC, resulting in TEG measurements not being systematically performed. A prerequisite for study inclusion was a pre-incision TEG, often missing in instances where TEG was requisitioned on demand during the surgery. Patients were considered eligible for the procedure if no extra-abdominal were identified in preoperative imaging, and if the functional status as graded by the Eastern Cooperative Oncology Group was ≤2. Patients were not taking anti-coagulation medications peri-operatively, except a single dose prophylactic s.c. Heparin given before surgery. The patients’ medical comorbidities were classified according to the Charlson comorbidity index (CCI). This study was approved by the Institutional Review Board (TLV-19-0463).

### CRS+HIPEC

CRS is comprised of both visceral resections and peritonectomies with the aim of resecting all macroscopic disease as described in detail elsewhere [15, 16]. The extent of peritoneal spread was classified according to the peritoneal cancer index (PCI) [16], and the completeness of cytoreduction (CCR) was used to measure residual disease at the end of the procedure [17]. HIPEC was performed by the closed technique for 90 min based on a mitomycin C regimen [18] for appendiceal, gastric and colorectal peritoneal metastases, and on a cisplatin and doxorubicin regimen for ovarian cancer [19]. Postoperative complications were graded according to the Clavien–Dindo (CD) classification [20], and SPC were defined as CD ≥3.

### Thromboelastogram (TEG) measurement

The first TEG was measured after anesthesia induction and before skin incision. The second TEG was obtained at CRS completion and before HIPEC initiation. The blood sample for TEG was collected into a citrated blood tube for coagulation. One milliliter of citrated whole blood was gently mixed with kaolin, and 360 μL of this preparation was pipetted into a TEG cup prewarmed to 37 °C and containing 20 μL calcium chloride. Measurements were performed in a TEG Hemostasis Analyzer 5000 (Haemonetics, Braintree, MA), which was calibrated daily by means of the controls supplied by the manufacturer before running the study samples. Analysis of TEG parameters was performed by TEG analytical software. The following TEG parameters were analyzed:

– R-time: the time interval from the beginning of the test until initial fibrin formation.
– K-time: the time interval until a 20-mm amplitude has been achieved on the graph.
– α Angle: the rate of clot formation.
– Maximum amplitude (MA): the strength of the fibrin clot (fibrinogen and platelets contribute 20 and 80 % of clot strength, respectively).
– LY30: the percentage decrease in graph amplitude 30 min after MA has been achieved. The LY30 measures the fibrinolytic system.

### Statistical analysis

Descriptive statistics for normally distributed parameters were given as means and standard deviations (SD), and medians and ranges were used for non-normally distributed parameters. Medians were compared by the Wilcoxon’s non-parametric test. Categorical values were presented by n (%) and compared by the chi-squared test. Multivariate logistic regression analysis was conducted for the factors found to be univariably associated with SPC. A p-value of <0.05 was considered significant. All statistical analyses were performed with SPSS for Windows version 27 (SPSS, Munich, Germany).

## Results

### Patients and procedure characteristics

Table 1 describes clinical and pathological characteristics of patients included in our study. We classified patients into two groups by SPC, defined as complications with CD ≥3. The complications encountered in our patient cohort were: small bowel obstruction (n=1), gastrointestinal leaks (n=4) and cardiac arrhythmia necessitating ablation (n=1). Patients with SPC did not differ from those without SPC regarding age, sex, tumor type, and preoperatively chemotherapy. The SPC group tended to have more medical comorbidities, although this tendency did not reach statistical significance. (median CCI [interquartile range, IQR]: 8 (6–8.25) vs. 7 (6–8), p=0.15). There were no statistically significant differences between groups in pre-operative laboratory values such as hemoglobin, white blood cell count, INR or albumin.

**Table 1:** Clinical characteristics of the study participants.

	All patients (n=37)	Patients w/o severe postoperative complications (n=31)	Patients with severe postoperative complications (n=6)	p-Value
Age, median (IQR)	55 (47–65)	55 (48–66)	55 (36.5–66)	0.94
Gender, F/M, %	24/13 (64.9/35.1)	20/11 (64.5/35.5)	4/2 (66.7/33.3)	0.92
Charlson comorbidity index, median (IQR)	7 (6–8)	7 (6–8)	8 (6–8.25)	0.15
Histology, n, %				0.49
Colon	19 (51.4)	11 (40.7)	5 (83.3)	
Ovary	6 (16.2)		0	
Appendix	4 (10.8)	2 (7.4)	1 (20)	
Meshothelioma	2 (5.4)	2 (7.4)	0	
Stomach	1 (2.7)	1 (3.7)	0	
Other	5 (13.5)	5 (18.5)	0	
Preoperative chemotherapy n, %	20 (54.1)	16 (51.6)	4 (66.7)	0.49

IQR, interquartile range.

Table 2 details the procedures performed in the two groups. Tumor and surgical extent did not differ between the groups according to the PCI, CCR, number of anastomoses and number of organs that were resected. Packed red blood cell transfusions were given to 9/31 (29 %) of the patients without SPC vs. 1/6 (16.6 %) of those with SPC (p=0.65). In addition, the two groups did not differ in estimated blood loss or in the number of the transfused red blood cell units, so that possible changes in TEG values (see below) were not related to transfusion requirements. As expected, patients with SPC had longer hospital stay than those without [median (IQR): 24.5 (19.5–54.5) vs. 13 (10–20), p=0.001].

**Table 2:** Procedure characteristics.

	All patients (n=37)	Patients w/o severe postoperative complications (n=31)	Patients with severe postoperative complications (n=6)	p-Value
PCI, median (IQR)	9 (4.5–12.5)	9 (5–14)	9 (3–10.5)	1
CCR, n, %				
0	32 (86.5)	26 (83.1)	6 (100)	0.73
1	2 (5.4)	2 (6.5)		
2	3 (8.1)	3 (9.6)		
No. of organs resected, median (IQR)	2 (1–3)	2 (1–3)	2 (0.75–2.25)	0.88
No. of anastmoses, median (IQR)	1 (0–1)	1 (0–1)	1 (0–1)	0.97
Estimated blood loss, median (IQR)	75 (0–237.5)	75 (0–300)	50 (0–200)	0.46
PC transfused, median (IQR)	0 (0–1)	0 (0–0.5)	0 (0–1)	0.71
LOS, median (IQR)	16 (10–23)	13 (10–20)	24.5 (19.5–54.5)	0.001

PCI, peritoneal carcinomatosis index; CCR, completeness of cytoreduction; IQR, interquartile range; LOS, length of hospital stay.

### TEG values after CRS showed hypocoagulable state in patients with SPC

Table 3 depicts the TEG values before and after the completion of CRS. Note that pre-operative TEG values were not different between patient with and without SPC, indicating that groups are initially comparable. The post-resection R-time (which measures the time to initial fibrin formation) and K-time (which measures clot strengthening and the rapidity of fibrin build-up) were prolonged in patients with SPC compared to those without SPC (6 ± 3.89 vs. 4.05 ± 1.24 s, p=0.01 and 1.65 ± 0.63 vs. 1.25 ± 0.42 s, p=0.03, respectively).

**Table 3:** Comparison of thromboelastogram (TEG) values before and after cytoreductive surgery (CRS).

Mean ± SD	Pre-operative TEG			Post-CRS TEG		
	Patients w/o severe postoperative complications (n=27)	Patients with severe postoperative complications (n=5)	p-Value	Patients w/o severe postoperative complications (n=27)	Patients with severe postoperative complications (n=5)	p-Value
R	4.97 ± 2.07	5.22 ± 1.93	0.89	4.05 ± 1.24	6 ± 3.89	<0.001
K	1.25 ± 0.41	1.55 ± 0.65	0.1	1.25 ± 0.42	1.65 ± 0.63	0.24
α	71.06 ± 12.93	71.45 ± 9.15	0.58	73.61 ± 5.42	68.67 ± 5.53	0.86
MA	67.28 ± 5.94	63.83 ± 5.17	0.41	65.65 ± 6.32	62.45 ± 4.28	0.46
LY30	0.35 ± 0.54	0.55 ± 0.95	0.11	0.35 ± 0.69	0.65 ± 0.86	0.33

### Change in TEG values is not associated with surgical or disease extent parameters

**Figure 1:**
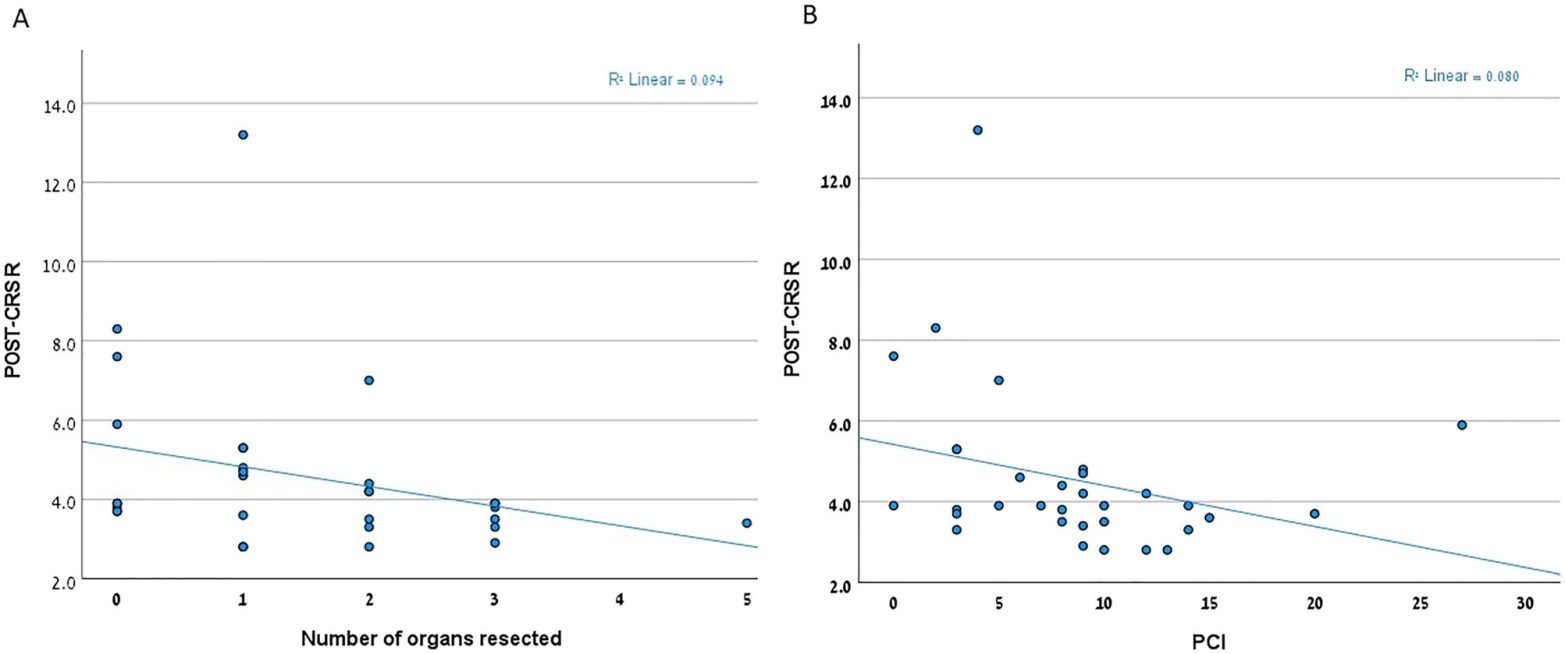
Association between the post-cytoreductive surgery (POST-CRS) R-time and surgery. (A) Number of organs that were resected; (B) peritoneal carcinomatosis index (PCI).

To determine whether TEG values are a genuine biomarker for postoperative complications, we wanted to see their correlations with other predictors of post-operative surgical complications, such as number of organ resected and tumor intra-abdominal extent (measured as PCI). Figure 1 displays the absence of any correlation between post-resection R-time values and the number of organs that had been resected (Figure 1A, r^2^=0.094), as well as the PCI (Figure 1B, r^2^=0.08). Thus, it seems that change in R value cannot be predicted by surgical or disease extent. Along the same line, Table 4 depicts uni- and multivariable analysis of factors associated with SPC. Since only a prolonged R-time and increased medical comorbidities (as measured by the CCI) were univariably associated with SPC in our cohort, we included those factors in a multivariate logistic regression in which only the post-resection R-time value retained its significance (odd ratio [OR]=1.53, p=0.05).

**Table 4:** Multivariable analysis of factors associated with severe post-operative complications.

	Univariate analysis		Multivariate analysis	
	OR	p-Value	OR	p-Value
Age	0.98	0.53		
CCI	1.22	0.13	1.41	0.36
Number of organs resected	0.94	0.86		
R value	1.47	0.04	1.53	0.05

## Discussion

SPC pose a major concern for patients with peritoneal surface malignancies who undergo CRS-HIPEC. In addition to the morbidity and subsequent impairment in their quality of life [21], SPC also increase the economic burden of healthcare [22]. Currently, there are no specific predictors of SPC, and clinical (age, medical comorbidities, sarcopenia, etc.) and procedure-related (tumor and resection extent, transfusion requirement, etc.) characteristics are used but with limited efficacy [13, 23]. The main findings of this study is that change in TEG values and, specifically, prolonged R- and K-times, were significantly associated with postoperative complications. A possible advantage of TEG values are their use for coagulation monitoring in these procedures and their rapid dynamics as opposed to CRP and albumin, that are changed in matter of days after surgery and cannot serve as intra-operative markers.

Although 50 % of the patients in our cohort were treated preoperatively with systemic chemotherapy, that treatment was not associated with increased morbidity in our series as well as in a recent report [24]. Interestingly, the extent of surgery, as measured by PCI and the number of organs that had been resected, was not associated with SPC. In fact, clinical experience also demonstrates the difficulty to predict SPC based on surgical extent, due to variability of patient response to surgical insult. Along the same line, in our study, the change in TEG values was not associated with surgical extent parameters such as PCI and number of organs resected. Hence, we suggest that TEG values measure an individual’s response to surgical stress.

In our study, patients without SPC had decreased R values after CRS, similar to another pilot study which reported the same trend after CRS on 15 patients [25]. The same study showed that HIPEC further decreased the same values, a measurement not taken in ours. Importantly, this study did not analyze TEG of patients with SPC, hence a direct comparison between our findings in patients with SPC is impossible.

The hypercoagulable state created by major surgery can be explained by the exposure of TF in the sub-endothelial basement membrane after endothelial damage and its interaction with factor VIIa and activation of thrombin [26]. Similarly, hypercoagulative state was also reported for pancreaticoduodenectomy [27]. The prolongation of TEG parameters and hypocoagulative state in patients with SPC merits further research, as it was not explained by consumption and is probably related to inflammation.

The association between change in TEG parameters and SPC during surgery may have several implications. As the number of intensive care beds is limited, it is important to avoid unnecessary admission which also increase hospital stay and costs [28] on the one hand, and an unplanned admission, which associated with increased mortality [29], on the other. Another implication of this association is related to stool diversion. There is an ongoing debate on the need and indications of stool diversion in CRS+HIPEC. Factors such as the number of anastomoses, their location and previous bevacizumab therapy were all studied for their association with anastomotic leaks with varying results 30], [31], [32. Our institutional policy is to divert stool with more than two anastomoses. However, the possibility to adapt the decision to divert stool according to a biomarker which is associated with SPC can improve surgical decision making. Importantly, to guide surgical and placement decisions, establishing a specific TEG change cut-off will require a larger patient sample. Our findings suggest that, to detect a mean difference of 1.95 in the post-CRS R value between groups, with a 30 % rate of SPC, a sample of 149 patients is needed (114 without SPC and 35 with SPC) to achieve a power of 0.8. Recruiting such a number of patients in a reasonable timeframe necessitates a prospective multi-institutional study. Therefore, our study should be viewed as hypothesis-generating.

While several risk prediction scores for morbidity and mortality, such as NSQIP, APACHE-II, and POSSOM [33–35], have been validated, none has been specifically validated in the context of CRS+HIPEC. A pertinent inquiry would be the comparison of these scores’ performance with TEG parameters in a prospective study setting. However, TEG’s advantage lies in its integration into the standard workflow for coagulation monitoring, in contrast to these scores, which require integration into clinical practice. Our study has several limitations. First, the relatively small number of patients with pre- and postoperative TEG results limits the applicability of our findings. However, the TEG results were the only marker associated with SPC even in this small number of patients. Second, we did not measure the TEG parameters after the HIPEC and did not correlate them to SPC. Although we found a significant correlation between post-cytoreduction TEG prolongation and complication, it is possible that HIPEC may also affect these values and contribute to the overall complication risks. It would be interesting to measure the individual contribution of CRS and HIPEC to changes in TEG parameters. However, it is commonly accepted that the CRS component of the procedure is has the largest contribution for the development of SPC after the procedure. In addition, as the use of HIPEC with the closed technique after anastomoses performance is common, and also practiced in our institution, we wanted to develop a tool that may assist intraoperatively, before abdominal closure.

To conclude, changes in TEG parameters can are associated with SPC, possibly due to their ability to quantify the inflammatory change induced by surgery. If they are validated as a viable SPC marker in a larger series, TEG parameters could assist in intraoperative decision making (such as diversion), predict patient prognosis and triage the intensity of postoperative care.
